# Homeostatic Plasticity Studied Using In Vivo Hippocampal Activity-Blockade: Synaptic Scaling, Intrinsic Plasticity and Age-Dependence

**DOI:** 10.1371/journal.pone.0000700

**Published:** 2007-08-08

**Authors:** Julio Echegoyen, Axel Neu, Kevin D. Graber, Ivan Soltesz

**Affiliations:** 1 Department of Anatomy and Neurobiology, University of California at Irvine, California, United States of America; 2 Department of Neurology and Neurological Sciences, Stanford University, Stanford, California, United States of America; Duke University, United States of America

## Abstract

Homeostatic plasticity is thought to be important in preventing neuronal circuits from becoming hyper- or hypoactive. However, there is little information concerning homeostatic mechanisms following in vivo manipulations of activity levels. We investigated synaptic scaling and intrinsic plasticity in CA1 pyramidal cells following 2 days of activity-blockade in vivo in adult (postnatal day 30; P30) and juvenile (P15) rats. Chronic activity-blockade in vivo was achieved using the sustained release of the sodium channel blocker tetrodotoxin (TTX) from the plastic polymer Elvax 40W implanted directly above the hippocampus, followed by electrophysiological assessment in slices in vitro. Three sets of results were in general agreement with previous studies on homeostatic responses to in vitro manipulations of activity. First, Schaffer collateral stimulation-evoked field responses were enhanced after 2 days of in vivo TTX application. Second, miniature excitatory postsynaptic current (mEPSC) amplitudes were potentiated. However, the increase in mEPSC amplitudes occurred only in juveniles, and not in adults, indicating age-dependent effects. Third, intrinsic neuronal excitability increased. In contrast, three sets of results sharply differed from previous reports on homeostatic responses to in vitro manipulations of activity. First, miniature inhibitory postsynaptic current (mIPSC) amplitudes were invariably enhanced. Second, multiplicative scaling of mEPSC and mIPSC amplitudes was absent. Third, the frequencies of adult and juvenile mEPSCs and adult mIPSCs were increased, indicating presynaptic alterations. These results provide new insights into in vivo homeostatic plasticity mechanisms with relevance to memory storage, activity-dependent development and neurological diseases.

## Introduction

Activity-dependent Hebbian plasticity, such as long-term potentiation (LTP) and long-term depression (LTD), is thought to have an inherently positive feed-back component that tends to destabilize neuronal networks [1]–[3]. However, there is now evidence that neuronal circuits possess various homeostatic plasticity mechanisms that counteract the destabilizing effects of Hebbian plasticity and ensure that neurons operate within a physiologically appropriate dynamic range. Specifically, studies from both invertebrates and vertebrates show that neurons are able to regulate their synaptic strengths and intrinsic neuronal properties in response to imposed changes in activity, in a manner that is consistent with homeostasis [4]–[12]. Homeostatic changes act to normalize overall neuronal firing after LTP or LTD occurring at individual synapses, and homeostatic rules have been proposed to play various functional roles, including improving signal propagation and the generation of self-organizing cortical maps [13]–[15]. Homeostatic plasticity mechanisms may also be engaged during abnormal activity patterns in neurological diseases, particularly in epilepsy [16]–[18], but the exact nature of the beneficial or deleterious roles such homeostatic processes may play in hyperexcitable disease states is only beginning to be elucidated.

Experimentally, neural activity can be artificially increased or decreased for prolonged periods of time, for example, by elevation of extracellular K^+^ concentration or by blocking action potentials with TTX [8]. It has been shown that visual cortical neurons in culture respond to decreased levels of activity imposed by prolonged TTX application with scaling up of miniature excitatory postsynaptic current (mEPSC) amplitudes and scaling down of miniature inhibitory postsynaptic currents (mIPSCs) [8], [19], [20]. Conversely, increases in activity result in synaptic changes in the opposite direction [8]. In addition to bidirectional changes in synaptic inputs, neurons can also modify their intrinsic neuronal properties and become hyperexcitable in response to TTX treatment [6]. Experimentally imposed decreases in activity in cultured hippocampal cells tend to lead to generally similar alterations as in cultures of the visual cortex, including neuronal hyperexcitability, increased glutamatergic transmission and decreased GABAergic synaptic inputs [4], [7], [16], [19], [21], [22], indicating the generality of homeostatic responses. Such activity-dependent changes are interpreted to be homeostatic because the direction of the alterations are such that they appear to counteract the imposed change in activity, resulting in stabilization of firing rates within the appropriate ranges [2].

However, the pre- or postsynaptic locus of the synaptic alterations triggered by changes in activity levels is less clear. Blockade of activity in cultures has been reported to lead to increases in mEPSC amplitude but not in frequency [8], [23]–[25], or increases in frequency but not in amplitude [4], or increases in both [7], [17]. Although the exact reasons for these apparent contradictions are not yet clear, the developmental stage of the tissue may be important [7], [26], [27]. For technical reasons having to do with the relative ease of manipulation of activity levels in vitro, most previous studies involving externally imposed alterations of activity were performed on developing neurons in culture systems. Although there had been a handful of studies on the cellular and synaptic processes set into motion by manipulations by activity levels in vivo [16], [28]–[30] there are no comprehensive investigations on whether and how in vivo homeostatic plasticity processes may be engaged by experimental changes in activity in neuronal circuits.

In order to investigate whether homeostatic plasticity occurs in the adult rat hippocampus in vivo, we modified a TTX delivery protocol [31] that consists of implanting wafers of the plastic polymer Elvax 40W loaded with TTX above the hippocampal CA1 area. This arrangement offers a local, long lasting delivery of TTX in vivo. After 48 hours of TTX application (a time often used in vitro studies of homeostatic plasticity; [6], [8]), we performed assessments of miniature excitatory and inhibitory synaptic currents and intrinsic neuronal properties in acute in vitro hippocampal slices taken from just below the implanted Elvax wafer. Our results revealed robust effects of in vivo manipulation of neuronal activity in the hippocampus on synaptic and intrinsic properties. However, several of the alterations were different both in their directions and in their loci (e.g., pre- versus postsynaptic) from previous reports that employed in vitro activity-blockade. The results also showed that the activity-dependent rescaling of synapses following in vivo manipulations did not occur in a multiplicative manner. In addition to focusing on the adult hippocampus, parallel experiments were conducted in juvenile hippocampi aimed at determining the importance of age-dependent responses to prolonged changes in vivo activity levels. Taken together, the current findings offer new insights into mechanisms underlying synaptic gain control, with particular relevance to the development of limbic epilepsy following insults such as trauma-induced deafferenation and the appearance of post-traumatic spontaneous seizures [18], [32].

## Methods

All protocols were approved by the Institutional Animal Care and Use Committee of the University of California, Irvine.

### TTX-Elvax preparation

Previously described techniques of preparation of Elvax were used [31]. Briefly, 2 mg TTX (Tocris, Ellisville, MO) was mixed dry with 100 mg of washed Elvax 40W and 1 ml methylene chloride. TTX was evenly suspended by vigorous mixing and the vial was then quickly frozen in a dry ice/ethanol bath. Methylene chloride solvent was allowed to slowly evaporate for 7 to 10 days at −20°C. The resulting cylinder of Elvax was sectioned using a Vibratome into 200 µm thick slices. Slices were stored at −20°C and washed at room temperature for 48 hours in saline before implantation to avoid the initial, fast phase of release that occurs before a slow, sustained release phase lasting at least 12 days [33], [34].

### Implantation of Elvax

We modified a formerly described technique [31] to perform a cortical undercut that allows for the placement of Elvax wafers above the CA1 area of the hippocampus. Wistar rats (P30 and P15; Charles River, Boston, MA) were anesthetized with a ketamine/xylazine (60 mg/Kg ketamine; 10 mg/Kg xylazine) mix and placed in a stereotaxic frame. The scalp was incised and a rectangular hole was trephined in the left parietal bone. The dura was then incised with fine scissors and reflected to expose the cerebral cortex. A micromanipulator was used to guide the position of a 25-gage needle bent 90°, 2 mm from the tip. The needle was inserted into the cortex at coordinates from bregma −1.0, lateral 0.5. The needle was introduced 1 mm into the cortex then the needle was advanced caudally 2 mm and retracted. After the needle was retracted, the resulting cortical slab was lifted and a 1mm by 1mm wafer of TTX-loaded Elvax (TTX Elvax), or control Elvax with no TTX (Control Elvax), was placed at coordinates from bregma −2.0, lateral −2.0. The wound was then closed and sutured. The animals were placed on a heating pad for recovery and returned to their cage upon awakening. Implantation was performed 48 hours before recordings. No obvious behavioral effects were observed after recovery from the implantation surgery.

### Slice preparation

Forty eight hours after Elvax implantation, coronal brain slices (450 µm for blind recordings, 350 µm for visualized recordings) were prepared as previously described [35], [36]. The animals were anesthetized with halothane, decapitated and their brains were removed. Slices were obtained from the area below the Elvax placement. The slices were incubated at 32°C in oxygenated (95% O_2_/5% CO_2_) artificial cerebrospinal fluid (ACSF; in mm 126 NaCl, 2.5 KCl, 26 NaHCO_3_, 2 CaCl_2_, 1.25 NaH_2_PO_4_, and 10 glucose) in a holding chamber for at least 2 hours prior to recording in order to allow complete washout of TTX.

### Electrophysiology

For extracellular recordings, individual slices were transferred to an interface-type chamber perfused with oxygenated ACSF at 36°C. Recording pipettes were filled with ACSF and placed in the CA1 pyramidal cell layer. To evoke field responses, constant-current stimuli (1–8 mA, 50–200 ms) were applied at 0.1 Hz using a 90 µm bipolar tungsten electrode positioned in the CA1 stratum radiatum. The placement and distance of the recording and stimulating electrodes were kept constant as described previously [37].

Blind whole-cell recordings were used for recordings of miniature excitatory postsynaptic currents (mEPSCs) and miniature inhibitory postsynaptic currents (mIPSCs). For the blind patch recordings, individual slices were transferred to an interface-type chamber perfused with oxygenated ACSF at 36°C. The ACSF, depending on the experiment, contained the following drugs: for mEPSCs, 10 µM bicuculine methchloride and 1 µM TTX; for mIPSCs, 10 µM D-2-amino-5phosphovaleric acid (APV), 10 µM 1,2,3,4-Tetrahydro-6-nitro-2,3-dioxo-benzo[f]quinoxaline-7-sulfonamide (NBQX), 1 µM TTX. Whole cell recordings were obtained from CA1 pyramidal cells with patch pipettes (4–7 MΩ) filled with internal solutions consisting of (in mM): for mEPSCs, 140 K-gluconate, 10 HEPES, 2 MgCl_2_, pH 7.20 (265–275 mOsm); for mIPSCs, 140 CsCl, 10 HEPES, 2 MgCl_2_, pH 7.10 (265–270 mOsm). Recordings were obtained with an Axopatch 200A (Axon Instruments, Foster City, CA) and digitized at 10 kHz. Visualized whole-cell recordings were used to measure alterations in intrinsic properties. For visualized whole-cell recordings, slices were transferred to a submerged-type recording chamber perfused with oxygenated ACSF at 33°C. Slices were visualized with an upright microscope (BX-50; Olympus, Tokyo, Japan) with infrared differential interference contrast optics. Whole-cell recording were obtained from visually identified (based on size and shape of cell body and primary dendrites) CA1 pyramidal cells with patch pipettes (3–5 MΩ) filled with internal solution containing (in mM): 126 K-gluconate, 4 KCl, 10 HEPES, 4 ATP-Mg, 0.3 GTP-Na and 10 phosphocreatine, pH7.2 (270–290 mOsm). Recordings were made using a MultiClamp 700A amplifier (Molecular Devices, Union City, CA). From a holding potential −60 mV, a series of depolarizing current pulses were applied at 20 pA steps and the number of action potentials was recorded. Input resistance was determined by −20 pA steps from a holding potential of −60 mV and calculated offline.

### Data Analysis

Recordings were digitized at 10 kHz with a Digidata 1322A analog-digital interface (Molecular Devices, Union City, CA). Statistical analyses were performed using Sigmaplot or SPSS, using a non-paired t-test or the non-parametric Kolmogorov-Smirnov test with a significance level of p<0.05. Results are described as mean±SEM.

For the cumulative probability plots, equal number of events were used from each cell (n = 20 for mEPSCs ; n = 100 for mIPSCs). For analysis concerning the presence or absence of multiplicative scaling [8], the control event amplitudes were ranked and then plotted against the ranked event amplitudes after TTX treatment, and the slope of the best linear fit was determined. The post-TTX event amplitudes were then divided by the scaling factor (i.e., the slope), and the Kolmogorov-Smirnov test was used to determine if the scaled-back post-TTX event amplitudes were significantly different from the control amplitudes. If they were significantly different, the increase in event amplitude could not be considered multiplicative.

## Results

### Hyperexcitable field responses in the CA1 region two days after implantation of TTX Elvax

**Figure 1:**
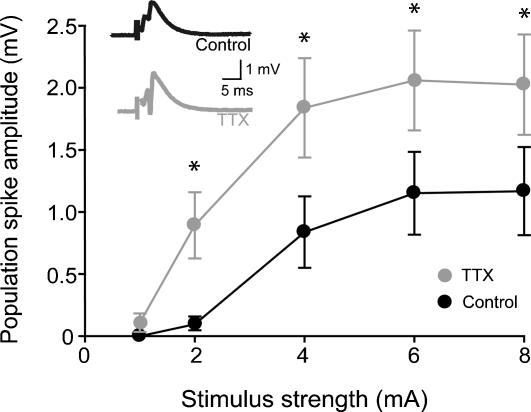
Hyperexcitability in the CA1 region of the hippocampus after in vivo activity-blockade. Forty-eight hours after implantation of the control or TTX-containing Elvax polymer, extracellular field potentials were recorded from the CA1 region in acute hippocampal slices in response to low-frequency electrical stimulation of the stratum radiatum. The figure shows summary data of the population spike amplitudes from TTX-treated and control rats (for number of slices and animals, see text), with example traces in the inserts.

First, we examined the effects of in vivo TTX treatment on the general excitability of the CA1 region in adult rats. Two days after Elvax implantation, extracellular field potentials were recorded from the CA1 pyramidal layer in acute slices, in response to electrical stimulation of the stratum radiatum at various stimulation intensities, and the population spike amplitude of the field excitatory post-synaptic potential (fEPSP) was measured, representing the synchronized firing of cells. As shown in Fig. 1, the population spike amplitude was significantly larger in slices from TTX Elvax implanted rats compared to control Elvax implanted rats at most stimulation intensities (fEPSPs were recorded in control ACSF; control: n = 8 slices, 4 animals; TTX: n = 9 slices, 4 animals).

Therefore, as expected from homeostatic plasticity responses, and generally in line with previous observations following 48 hours of TTX treatment in vitro [6], the CA1 region of the hippocampus became hyperexcitable following activity suppression in vivo. We hypothesized that this increase in hyperexcitability may be due to changes in synaptic strength and/or to alterations in the intrinsic properties of neurons. Therefore, in the subsequent experiments, we aimed to determine if synaptic and intrinsic parameters were altered after in vivo TTX treatment.

### In vivo activity-deprivation enhances excitatory inputs primarily through presynaptic mechanisms

**Figure 2:**
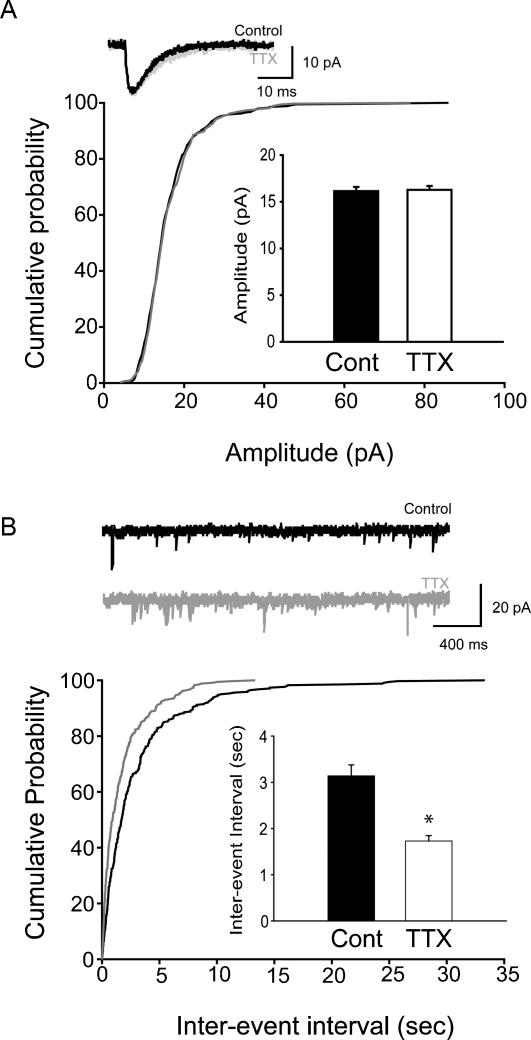
Unchanged amplitude, but increased frequency of mEPSCs in CA1 pyramidal cells from adult animals after in vivo TTX-application. (A) Summary data (from all cells) of the mEPSC amplitudes in TTX-treated and control adult rats are shown in the form of cumulative probability plots (for numbers of events and animals, see main text); the bar graph shows the same data in the form of averages±SEM, and the insert displays representative averages of mEPSCs from single cells. (B) Summary data of the inter-event intervals of mEPSCs, with example traces.

In order to investigate changes in synaptic strength, we first studied mEPSCs in CA1 pyramidal cells from adult rats. Contrary to what was expected based on previous studies in cultures [8], there was no TTX-induced change in the mEPSC amplitude (Control: 16.1±0.44 pA; TTX: 16.3±0.41 pA; Fig 2A; control: n = 10 cells, 5 animals; TTX: n = 10 cells, 5 animals) or kinetics (Table 1). However, a significant increase in the frequency of mEPSCs was observed (inter-event intervals: Control: 3.1±0.23 sec, TTX: 1.7±0.11 sec; Fig 2B; control: n = 17 cells, 5 animals; TTX: n = 17 cells, 5 animals).

**Table 1:** Kinetics of mEPSCs and mIPSCs recorded from adult and juvenile CA1 pyramidal cells 48 hours after implantation with control Elvax or TTX Elvax (asterisks indicate significant difference (p≤0.05)).

		mEPSCs		mIPSCs	
		ADULT	JUVENILE	ADULT	JUVENILE
Rise time 10–90% (ms)	Control	1.39±0.14	1.43±0.10	0.56±0.04	0.69±0.87
	TTX	1.57±0.90	1.41±0.10	0.43±0.03*	0.72±0.04
Decay time constant (ms)	Control	7.62±0.56	5.33±0.28	6.36±0.43	8.46±0.32
	TTX	6.87±0.36	5.63±0.26	4.66±0.27*	8.16±0.35

**Figure 3:**
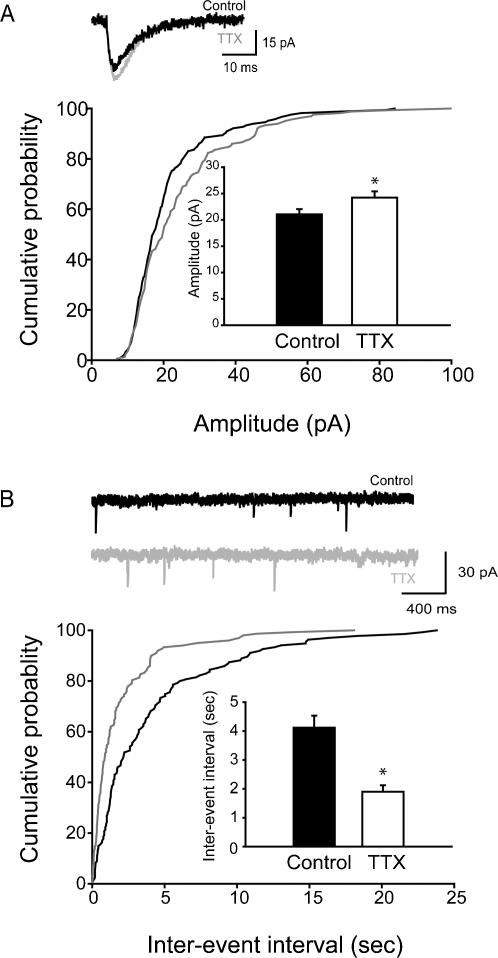
Increased amplitude and frequency of mEPSCs in juvenile CA1 cells after in vivo TTX-application. (A) Summary data of the mEPSC amplitudes; insert: representative averages from single cells. (B) Summary data of the inter-event interval of mEPSCs, with representative traces as inserts.

As there was no increase in the amplitude of mEPSCs in adult animals after in vivo TTX treatment, in subsequent experiments we investigated mEPSCs in juvenile animals (P15±2 days), because the latter age correlates more closely with the age of the neuronal cultures used in previous studies of homeostatic plasticity that reported significant alterations in mEPSC amplitudes following activity-blockade [4], [5], [7], [8], [28]. Indeed, in cells from slices from juvenile rats treated with TTX Elvax, there was a significant increase in the mEPSC amplitude (Control: 21.0±0.9 pA, TTX: 24.2±1.2 pA; Fig 3A; control: n = 10 cells, 6 animals; TTX: n = 10 cells, 5 animals), with no change in the kinetics (in Table 1). As in the adults, the mEPSC frequency was also increased in the juvenile rats after TTX treatment (inter-event intervals: Control: 4.11±0.41 sec, TTX: 1.9±0.22 sec; Fig 3B; control: n = 10 cells, 6 animals; TTX: n = 10 cells, 5 animals).

Taken together, these results suggested that activity deprivation, as expected from synaptic homeostasis, tended to increase excitatory inputs. However, the primary mechanism appeared to be an increase in frequency of events (representing presynaptic plasticity processes), and the expected enhancement in mEPSC amplitude was only found at a younger developmental stage, but not in the adult animal.

### In vivo TTX application increases mIPSC amplitude in both adults and juvenile rats

**Figure 4:**
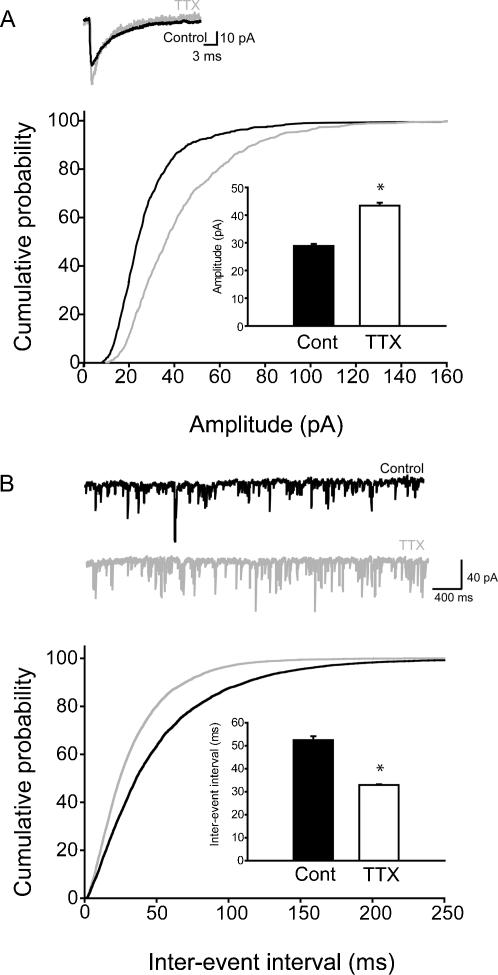
Increased amplitude and frequency of mIPSCs in adult CA1 cells after in vivo TTX-application. (A) Summary data of the mIPSC amplitudes (for numbers of cells and animals, see main text); insert: representative averages from single cells. (B) Summary data of the inter-event interval of mIPSCs, with representative traces.

In addition to the increased excitatory inputs, a reduction in inhibitory currents may also contribute to the increase in population spikes in the CA1 area after in vivo TTX treatment (Fig. 1). In agreement with this possibility, mIPSCs have been reported to show a reduction in amplitude after TTX application in cultures [20], [38]. In order to determine how GABA_A_ receptor mediated synaptic inputs changed following in vivo TTX treatment, we recorded mIPSCs 2 days after TTX Elvax implantation in adult rats. Surprisingly, the mIPSCs recorded from adult rats after TTX treatment showed a significant increase in amplitude (Control: 28.9±0.72 pA, TTX: 43.43±1.05 pA; Fig 4A; control: n = 11, 4 animals; TTX: n = 8, 4 animals). In addition, the frequency of mIPSCs from adult rats was increased (Inter-event interval: Control: 52.39±1.67 ms, TTX: 32.86±0.32 ms; Fig 4B; control: n = 11, 4 animals; TTX: n = 8, 4 animals). The kinetics of the events also changed, as both the rise time and the decay time of the mIPSCs became faster (Table 1). The net change, resulting from a combination of the increased amplitude and frequency of faster individual events, was a general augmentation of GABA_A_ inputs to CA1 pyramidal cells, as represented by the significantly enhanced mIPSC charge transfer (control: 15.0±0.6 pA*ms; TTX: 20.5±0.8 pA*ms).

**Figure 5:**
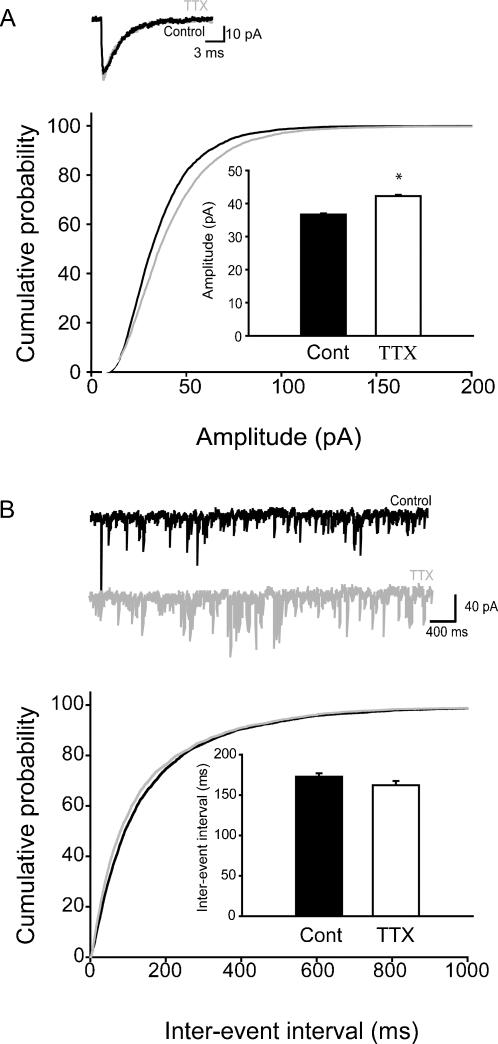
Increased amplitude, but no change in frequency, of mIPSCs in juvenile CA1 cells after in vivo TTX-application. (A) Summary data of the mIPSC amplitudes; insert: representative averages from single cells. (B) Summary data of the inter-event interval of mIPSCs, with representative traces.

Next, we tested whether the observed activity-blockade-induced scaling up of the mIPSCs was due to the age of the animals. Similar to the adult mIPSC data, the amplitude of the mIPSCs recorded from juvenile animals 48 hours after implantation with TTX Elvax was also significantly increased (Control: 36.86±0.30 pA, TTX: 42.20±0.41 pA; Fig 5A; control: n = 11 cells, 5 animals; TTX: n = 11cells, 6 animals). In contrast to the adults, however, there was no change in mIPSC frequency (inter-event intervals: Control: 172.69±4.24 ms, TTX: 161.91±5.39 ms; Fig 5B; control: n = 11 cells, 5 animals; TTX: n = 11cells, 6 animals), or kinetics (Table 1). Taken together, these data indicated that in vivo activity-blockade increased the amplitude of mIPSCs in both juvenile and adult hippocampi. However, as indicated by the differential responses to TTX treatment in mIPSC frequency between the juveniles and adults, activity-blockade also had age-dependent effects on GABAergic currents.

### Lack of multiplicative scaling of synaptic currents after activity-blockade in vivo

**Figure 6:**
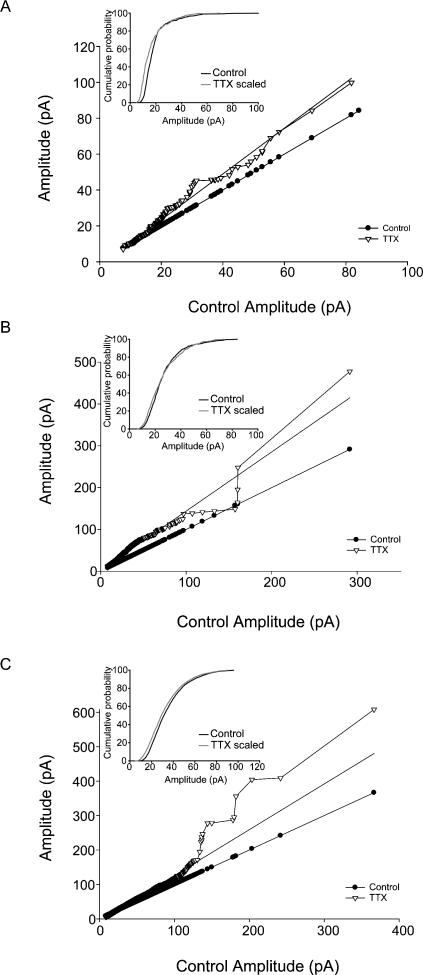
Lack of multiplicative scaling after in vivo TTX-treatment. (A–C) Plots of miniature events, ranked by amplitude, from TTX-treated versus control cells are shown; inserts: cumulative distribution plots of control amplitudes and scaled TTX events (for scaling details see text; scaling factors: juvenile mEPSCs = 1.27; adult mIPSCs = 1.22; juvenile mIPSCs = 1.33). (A) mEPSCs from juveniles (linear fit equation; TTX = 1.27(Cont)−2.14; R^2^ = 0.99). (B) mIPSCs from adults (linear fit equation; TTX = 1.22(Cont)+2.65; R^2^ = 0.95). (C) mIPSCs from juveniles (linear fit equation; TTX = 1.33(Cont)−6.77; R^2^ = 0.97). The scaled-back post-TTX event amplitudes (for details, see main text) remained significantly different from the control amplitudes, indicating lack of multiplicative scaling.

In some of the previous studies on homeostatic plasticity mechanisms in cultures, TTX treatment has been reported to result in a scaling up of mEPSC amplitudes by the same factor, revealing the presence of multiplicative scaling [8]. Multiplicative scaling implies that the cell regulates all of its inputs in a coordinated manner; for example, by increasing postsynaptic receptor efficacy or number at all active synapses by a certain percentage, regardless of initial amplitude. Therefore, we carried out additional analysis of the event amplitudes to determine the presence or absence of multiplicative scaling (see Methods). As described above, there were TTX-induced increases in miniature events in three of the four cases examined (juvenile mEPSCs, and juvenile and adult mIPSCs). As shown in Fig. 6, none of the enhanced synaptic inputs were multiplicatively, or proportionally, scaled following TTX treatment in vivo (note that the lack of multiplicative scaling is represented by the fact that, as illustrated in Fig. 6, the scaled-back post-TTX event amplitudes remained significantly different from the control amplitudes).

A previous report indicated the preferential involvement of very large synapses in excitatory event potentiation following activity-blockade with NBQX in cultured hippocampal neurons [7]. Therefore, we also tested whether the reason for the lack of multiplicative scaling was the presence of a few very large events that were scaled differently than the rest of the event population. However, there was no multiplicative scaling even after the exclusion of the largest 2% of the events (data not shown).

### Increased intrinsic excitability after in vivo TTX treatment in both adult and juvenile animals

**Figure 7:**
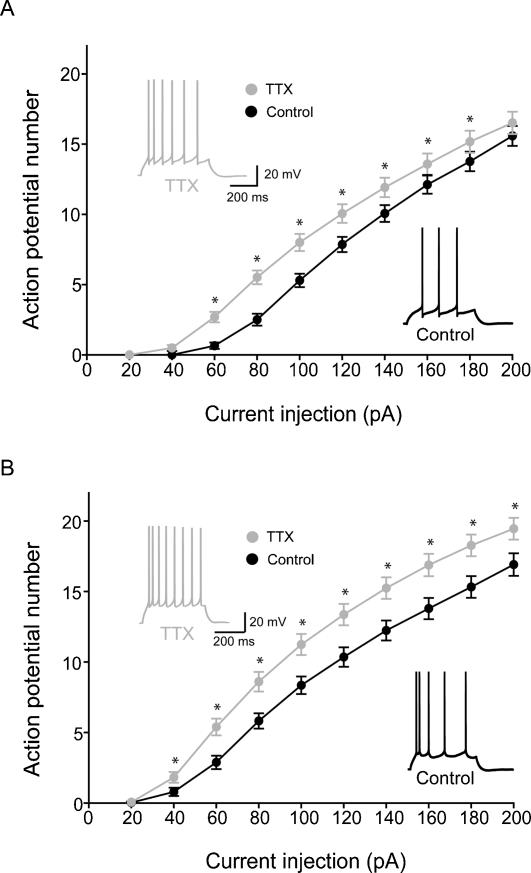
Increased intrinsic excitability in CA1 cells from adult and juvenile rats after in vivo TTX-application. (A,B) Summary plots of action potential numbers as a function of current injection (from −60 mV; duration: 500ms; for number of cells and animals, see main text) from adult (A) and juvenile (B) rats, with representative traces from single cells. Asterisks indicate significant (p<0.05) difference between the control and TTX groups.

The hyperexcitability observed in our field recordings in the CA1 region after TTX treatment may also be due to changes in the intrinsic properties of the pyramidal neurons [6]. Therefore, in the final set of experiments we measured several intrinsic parameters of excitability, including the input resistance, the number of action potentials evoked by depolarizing current injections (firing-input current or I-F curves), and resting membrane potential (V_m_) in CA1 pyramidal cell neurons from animals implanted with TTX Elvax or control Elvax. Forty-eight hours after TTX Elvax implantation, the input resistance of CA1 pyramidal cells was significantly increased in both adult and juvenile animals (Adult: Control: 114.4±2.5 MΩ, TTX: 133.6±4.3 MΩ; Control: n = 34, 5 animals; TTX: n = 35, 5 animals; Juvenile: Control: 136.1±4.3 MΩ, TTX: 174.1±5.7 MΩ; Control: n = 34, 5 animals; TTX: n = 33, 5 animals), without changes in resting membrane potential (Adult: Control: −64.9±0.5 mV; TTX: −66.3±0.6 mV; Juvenile: Control: −61.7±0.4; TTX: −62.2±0.4 mV). Importantly, both adult and juvenile neurons fired significantly more action potentials after TTX treatment in response to depolarizing current injections (Fig 7A&B; Control: n = 34 cells, TTX: n = 35 cells). These data showed that adult and juvenile CA1 neurons became more excitable as a result of alterations in their intrinsic properties following in vivo TTX treatment.

## Discussion

### Synaptic plasticity in the CA1 region in vivo

A main finding of this paper is that activity-deprivation in vivo results in alterations in synaptic properties that are often distinct from those observed following TTX application in culture systems. While the overall direction of the alterations in CA1 excitability, represented by the enhanced field responses, was in general agreement with what could be expected from homeostatic plasticity, the specific alterations in synaptic properties presented a considerably more complex picture. Nevertheless, certain general rules can be observed. First, the amplitudes of the mEPSCs and mIPSCs either increased or did not change in response to TTX treatment, but there were no instances where activity-blockade resulted in decreases in miniature event amplitudes. Second, the frequency of the mE/IPSCs showed a similar pattern, i.e., the mE/IPSC frequency either significantly increased or remained unchanged, but never decreased. Third, in every one of the four cases examined (mEPSCs and mIPSCs in adults and juveniles), there was some significant alteration elicited by the in vivo TTX treatment (namely, either increased event amplitude, or increased frequency, or both), indicating that both glutamatergic and GABAergic synapses respond to activity-blockade in the developing and adult hippocampi.

Therefore, activity-deprivation in vivo appeared to cause a general enhancement of synaptic inputs from both glutamatergic and GABAergic terminals. However, we saw no overarching, general rules concerning the pre- versus postsynaptic locus of the observed synaptic alterations. Previous reports on the effects of activity-blockade in cultures often showed increases in mEPSC amplitudes without concurrent changes in frequency [8], [23], [24]. However, there have been several studies that showed alterations in mEPSC frequency either with or without concurrent changes in amplitudes [4], [7], [22]. Structural studies also pointed to complex pre- and/or postsynaptic alterations [39]–[42]. A recent study suggested that the locus of plasticity concerning glutamatergic synapses may depend on the time in vitro, not on the actual age of the tissue[43]. Our data, showing increases in both amplitude and frequency in juvenile mEPSCs but only frequency-enhancements in adult mEPSCs, seem to suggest that the age of the animal may also play a role in the locus of the plasticity at hippocampal excitatory synapses in response to activity-deprivation.

An additional finding that was consistent across our data sets was the lack of multiplicative scaling for the enhanced synaptic inputs, even after the removal of the largest events [7]. The presence of multiplicative (or proportional) synaptic scaling, observed after manipulations of activity in cultures, suggested that homeostatic synaptic plasticity scales neuronal output without modifying the relative strength of individual synapses [2], [44]. It is not clear why this was not observed in our experiments with in vivo TTX treatment. However, our data are in general agreement with recent in vitro data indicating non-global adaptive mechanisms, where only a portion of the presynaptic axon terminals is affected by chronic manipulations in firing activity [42]. Potential explanations for the lack of multiplicative scaling may also include the larger number and more diverse synaptic inputs in the in vivo situation, which are at least partially preserved in acute slices. It is possible, and perhaps even likely, that inputs from different sources and cell types react differently to in vivo activity-deprivation.

The possibility that distinct cell types respond differently to changes in activity levels is an especially valid consideration for hippocampal GABAergic cells expressing characteristically high levels of heterogeneity, both in terms of the existence of separate interneuronal cell types (diversity) and in the form of cell to cell variability within an individual interneuronal class [45], [46]. However, homeostatic responses exhibited by GABAergic systems are not well understood. In vitro studies from cortical and hippocampal cells reported that activity-deprivation invariably resulted in decreases in mIPSC amplitude, either without [19], [20] or with [17], [38] concurrent decreases in event frequency. In contrast, our data showed increases in mIPSC amplitudes in response to in vivo TTX treatment in both adult and juvenile hippocampi. While we cannot resolve the reasons for the difference, it is interesting to note that cortical inhibition has been shown to undergo potent enhancement following visual deprivation in vivo, with a threefold increase in IPSCs between fast spiking cells (most likely basket cells) and pyramidal cells [30]. The observed speeding up of the mIPSC kinetics in the adult hippocampi observed in our experiments may be related to modifications in subunit expression, but it may also be due to differential effects of in vivo TTX-application on distinct interneuronal classes resulting in alterations in the cellular origin of the mIPSCs. Future studies will be needed to examine how different interneuronal types respond to various in vivo manipulations of activity.

### Altered intrinsic membrane properties in response to activity-deprivation

Although much attention has been focused on how chronic manipulations in activity levels affect synaptic scaling, it is now clear that another potential substrate for plasticity is the various intrinsic conductances expressed by neurons. Research on invertebrate neurons demonstrated that activity can regulate the expression of voltage-gated conductances [12], [31], [47]–[49]. Work on vertebrate neurons in cultures generally confirmed these observations [6], [21], [50]. In neocortical pyramidal cells, for example, prolonged blockade of activity has been shown to lead to a lowering of spike threshold, resulting in a higher firing frequency to current injections [6], primarily associated with increases in Na^+^ current and decreases in persistent K^+^ currents. There is also evidence that TTX treatment in hippocampal cultures enhances intrinsic bursting, resulting from upregulation of Ca^2+^ channels [25]. Our data from in vivo activity-blockade in the hippocampus, demonstrating enhanced input resistance and increased firing to depolarizing current inputs, generally agreed with these prior observations. In addition, our results showed similar alterations in both adult and juvenile hippocampi, in line with recent data indicating that plasticity of intrinsic excitability may be less sensitive to developmental stage [50]. Activity-dependent alterations in intrinsic currents may play a variety of functional roles, including the regulation of synaptic plasticity, gating of backpropagating action potentials, and modulation of the output properties of cells to match the properties of their inputs [51]–[54]. While plasticity of intrinsic excitability may not be invariably homeostatic [55], the direction of the changes in intrinsic excitability observed in this paper following in vivo blockade of activity in the hippocampus appeared to generally follow the homeostatic principle.

### Towards understanding the role of homeostatic plasticity in limbic epilepsy

In addition to contributing to various normal neuronal operations, homeostatic plasticity mechanisms may also play key roles in the emergence of epilepsy after insults [16]–[19], [25], [32]. A recent paper demonstrated that traumatic head injury in vivo resulted in long-lasting, robust changes in Na^+^, K^+^ and h-currents in mossy cells in the dentate gyrus [18]. Curiously, in spite of the presence of significant alterations, the current-voltage and current firing frequency curves remained unchanged in these cells, indicating the finely coordinated, apparently homeostatic nature of these alterations in intrinsic conductances after the insult. Although it is not yet clear how single cell homeostasis may function in a persistently hyperexcitable network (for a discussion, see [18]), it seems that homeostatic plasticity of both intrinsic excitability and synaptic inputs is a potentially key factor in the emergence and maintenance of spontaneous seizures following insults [32]. Our data in this paper demonstrate that synaptic and intrinsic properties undergo significant alterations in CA1 pyramidal cells after in vivo blockade of activity in the hippocampus. While the overall direction of the change in network excitability, as reflected by the enhanced field responses to stimulation of afferent pathways, was in general agreement with homeostasis, future research will be needed to establish how specific alterations in glutamatergic and GABAergic inputs and intrinsic conductances contribute to the hyperexcitability induced by the chronic activity-blockade. Finally, comparisons of results from future experiments with prolonged in vivo manipulations of activity in control and post-traumatic hippocampi are likely to contribute to our understanding of how homeostatic plasticity mechanisms may be engaged by insults to promote or counteract the emergence of post-traumatic epilepsy.
